# Contraceptive discontinuation among women of reproductive age in Papua New Guinea

**DOI:** 10.1186/s40834-022-00170-3

**Published:** 2022-06-02

**Authors:** Louis Kobina Dadzie, Abdul-Aziz Seidu, Bright Opoku Ahinkorah, Justice Kanor Tetteh, Tarif Salihu, Joshua Okyere, Sanni Yaya

**Affiliations:** 1 Department of Population and Health, University of Cape Coast, Cape Coast, Ghana; 2 Department of Estate Management, Faculty of Built and Natural Environment, Takoradi Technical University, Takoradi, Ghana; 3 Centre For Gender and Advocacy, Takoradi Technical University, Takoradi, Ghana; 4 College of Public Health, Medical and Veterinary Sciences, James Cook University, Townsville, QLD Australia; 5 School of Public Health, Faculty of Health, University of Technology Sydney, Sydney, Australia; 6 School of International Development and Global Studies, University of Ottawa, Ottawa, Canada; 7 The George Institute for Global Health, Imperial College London, London, UK

**Keywords:** Contraceptive Discontinuation, Family Planning, Papua New Guinea, Global Health

## Abstract

**Background:**

Papua New Guinea has one of the lowest contraceptive prevalence rates among women of reproductive age in the Western Pacific Region and this makes contraceptive discontinuation in this country a critical public health issue worth studying. This study sought to assess the factors associated with contraceptive discontinuation among women of reproductive age in Papua New Guinea.

**Methods:**

The data used for the analysis were obtained from the Papua New Guinea Demographic and Health Survey which was conducted in 2016–2018. The outcome variable for this study was contraceptive discontinuation among women of reproductive age. Crude odds ratios and adjusted odds ratios with 95% confidence intervals were estimated using binary logistic regression.

**Results:**

About 33.26% of the women discontinued injectables, 19.15% discontinued pills and 3.77% discontinued other contraception methods. Women aged 20–24 [aOR = 2.12, CI = [= [1.04,4.31] through to those aged 30–34 [aOR = 1.98, CI = 1.03,3.79] had higher odds to discontinue contraceptive usage compared to those aged 45–49. Women with no information on choice of contraception [aOR = 2.85, CI = 2.31,3.51], those with two or more births in the last five years [aOR = 2.35, CI = 1.65,3.35] and those living in the Highland region [aOR = 1.71, CI = 1.28,2.29] were more likely to discontinue contraceptive usage compared with those with information on contraceptive choices, those with no births and those living in the Island region respectively. However, women in the rural areas [aOR = 0.78, CI = 0.61,0.99], women using LARC [aOR = 0.10, CI = 0.06,0.15], injectables [aOR = 0.43, CI = 0.30,0.63] and other modern contraception methods including condom [aOR = 0.22, CI = 0.15,0.34] were less likely to discontinue contraceptive usage.

**Conclusion:**

A nationwide mass education on the benefits of contraception is recommended for the Papua New Guinea National Department of Health to tackle the key findings of this study which were high contraceptive discontinuation prevalence with lack of information on choice, disproportionately high contraceptive discontinuation rate in the Highland Region and the desire to give birth to more than two children as some factors associated with contraceptive discontinuation in Papua New Guinea.

## Background

Family planning (FP) is fundamental to achieving the Sustainable Development Goals (SDGs), particularly (SDG 3), which focuses on ensuring good health and wellbeing. Globally, it has contributed to reductions in unplanned pregnancies, unwanted pregnancies, maternal deaths and under 5 mortality [1, 2]. Access to quality sexual and reproductive healthcare services, including modern contraception is sturdily interconnected with women’s and children’s health, poverty, education, gender equality and human rights [2]. Women and girls who have access to contraceptives are more likely to realize higher socioeconomic status through education, employment, empowerment, and these quicken the progress of the country by reducing healthcare expenditures [3]. Lack of access and inconsistent use of contraception by women lead to majority of unintended pregnancies and unsafe abortions which eventually result in poor maternal and child health outcomes, affecting the progress of a country [3]. Hence, greater access to and consistent utilization of contraception are fundamental in the decline of unintended pregnancies and abortions.

In spite of women’s desire to delay or limit childbearing, contraceptive discontinuation presents a vital public health issue [4]. Contraceptive discontinuation indicates an imperative reproductive health concern that has an adverse consequence on women’s reproductive health outcomes [5]. A greater percentage of contraceptive discontinuation that happens when women do not wish to get pregnant is frequently linked with unintended pregnancies, unwanted births and unsafe abortions which have augmented menaces of pregnancy and childbirth-related maternal morbidity and mortality, as well as poor infant and child health outcomes [6, 7]. The effect of FP programs can be adversely influenced by contraceptive discontinuation which has consequences for economic growth.

Dynamics of contraception, including discontinuation, switching, and failure are significant indicators to detect gaps in public health programs on FP needs of women and couples [5]. In a Demographic and Health Survey (DHS) analysis carried out in 34 countries, the authors found that among women who had ever utilized a modern method, approximately 38% discontinued using a modern method, notwithstanding the ongoing desire for FP, and previous use accounted for more than 50% of all women with unmet need in 16 of the countries [8]. Additionally, it was indicated that unmet need was found to be high where contraceptive discontinuation is high and contraceptive prevalence is low [9]. Conversely, an analysis of DHS datasets in 14 countries reported that a significant number of women who had not switched to another contraceptive method remained at higher risk for pregnancy [10].

Several studies [11–13] have concentrated globally on identifying the reasons for discontinuation of diverse methods of contraception. The choice to continue or discontinue the use of contraceptives can be induced by a number of factors including, age of woman, number of living children (parity), desired number of children, having television and radios, decision-maker to use contraceptive, husband/ partner support, perceived benefit to the FP, perceived contraceptive harm, duration of contraceptive use, counseling on FP and contraceptive side effects, experience of side effects, access and availability of different type of contraceptive [7, 10, 11, 14]. In a study involving 36 low-and middle-income countries, the authors found that about 2% of unintended pregnancies in the preceding 5 years were because of discontinuation of traditional methods, 18% were because of discontinuation of short-acting modern methods, and 2% were because of long-acting methods.

Additionally, a study involving 25 low and middle-income countries indicated that an estimated 38% of women discontinued contraceptive use within 12 months, 55% within 24 months, and 64% within 36 months. Discontinuation rates within 12 months were greatest among condom users (50%) and lowest among intrauterine device (IUD) users [5], whereas about 40% of users of the pill, injectables, periodic abstinence, and withdrawal discontinued within 12 months. Discontinuation due to health concerns /side effects, a method related reason, was the most common reason for discontinuation [5]. Hence, minimizing early discontinuation among women presently utilizing contraception can be an operative approach in decreasing unmet need and unintended pregnancies [15].

Papua New Guinea is one of the countries in the western pacific region that still encounters substantial challenges in access to family planning services [16]. The country has a contraceptive prevalence rate of 20%-24% (rural–urban) [17] with unmet need for contraception at 25% [18]. This contributes to high maternal mortality ratios (MMR), estimated to be between 300 per 100,000 in urban settings and 900 per 100,000 in rural settings [18]. Due to this, the WHO has suggested consistent use of contraception, as one of the strategic approaches for reducing maternal mortality in the country [17, 19–21]. To help understand why national contraceptive roll out and programs in the past in PNG has not yet produced the best outcome, we sought to assess some factors associated with contraceptive discontinuation in PNG in order to inform policies and programs targeted at increasing family planning utilization among women of reproductive age.

## Methods

### Data source and study design

The study was a cross-sectional study that used data from Papua New Guinea Demographic and Health Survey (PGDHS), which was conducted in 2016–2018. The main aim of the PGDHS is to provide current information on basic demographic and health indicators such as contraception. Inner City Fund (ICF) provided technical assistance for the survey through the Demographic and Health Survey Programme. The provinces in the country of focus were further divided into 43 strata, paying attention to urban–rural differentials; however, the National Capital District did not have any rural strata. Each stratum provided samples of census units. The first stage involved the use of a probability proportional-to-size sampling. The second stage of the sampling involved the selection of 24 households from each of the clusters, using an equal probability systematic selection, with the resulting sample being about 19,200 households. About 17,505 households were selected for the sample, 16,754 of which were occupied and 16,021 of the occupied households were interviewed, with a response rate of 96%. From these households, 18,175 women of reproductive age were identified for individual interviews, with 15,198 women completing the interviews at a response rate of 84%. The sample used in this study was 4362. Details of the methodology, pretesting, training of field workers, the sampling design, and selection are available in the PGDHS final report which is also available at https://dhsprogram.com/ publications/publicationfr364-dhs-final-reports.cfm. We relied on the “Strengthening the Reporting of Observational Studies in Epidemiology” (STROBE) statement in writing the manuscript.

### Variables

#### Outcome variable

The outcome variable for this study was contraceptive discontinuation among reproductive-age women (15–49 years). This was measured as the percentage of reproductive-age women who used a method of contraception in the last 5 years prior to the survey, but discontinued using within 12 months after beginning to use it.

#### Explanatory variables

Age, marital status, educational level, place of residence, occupation, wealth index, region, exposure to family planning messages, knowledge on family planning, information on choice of contraception, decision maker for using contraception, births in last five years, number of living children, desire for more children and type of contraceptive method comprised the explanatory variables (See Table 1). These variables were informed by previous studies and a priori [4, 22, 23].

**Table 1:** Socio-demographic, reproductive and obstetric characteristics of reproductive age women in Papua New Guinea, 2016-2018 PGDHS (weighted)

Variable	Freq	Percent
**Age in 5-year groups**		
15–19	137	3.1
20–24	811	18.6
25–29	1,124	25.8
30–34	1,066	24.4
35–39	751	17.2
40–44	344	7.9
45–49	129	3.0
**Marital status**		
Living together	553	12.7
Married	3,437	78.8
Never married/widowed/divorced/separated	371	8.5
**Highest educational level**		
No education	686	15.7
Primary	2,220	50.9
Secondary	1,212	27.8
Higher	244	5.6
**Place of residence**		
Urban	700	16.0
Rural	3,662	84.0
**Occupation**		
Not working	2,694	61.8
Working	1,668	38.2
**Wealth index**		
Poorest	563	12.9
Poorer	741	17.0
Middle	872	20.0
Richer	1,046	24.0
Richest	1,141	26.2
**Region**		
Southern region	1,071	24.6
Highlands region	1,518	34.8
Momase region	1,071	24.5
Islands region	702	16.1
**Exposure to family planning messages**		
No	3,312	75.9
Yes	1,050	24.1
**Knowledge of family planning**		
Poor	914	21.0
Good	3,448	79.0
**Information on choice**		
No	2,439	55.9
Yes	1,923	44.1
**Decision maker for using contraception**		
Mainly respondent	809	18.6
Mainly husband/partner	435	10.0
Jointly	2,042	46.8
Others	1,076	24.7
**Births in the last five years**		
None	650	14.9
1	2,129	48.8
2 +	1,583	36.3
**Number of living children**		
None	192	4.4
1	711	16.3
2	858	19.7
3	927	21.3
4 +	1,674	38.4
**Desire for more children**		
Wants within 2 years	199	4.6
Wants after 2 + years	824	18.9
Wants no more children	1,694	38.8
Undecided	1,645	37.7
**Method**		
LARC	1,067	24.5
Pills	469	10.7
Injectables	1,262	28.9
Other modern methods including condom	751	17.2
Traditional method	813	18.6

*LARC* Long Acting Reversible Contraceptive

### Analysis

Data were extracted from the women’s file and cleaned. We made use of the DHS calendar file and created the event file in order to analyze the contraceptive discontinuation events among women aged 15–49 who used contraception in 5 years before the survey and discontinued within 12 months after beginning its use. Discontinued events was the unit of analysis for further exploration. Frequency counts and percentages were estimated for categorical variables. Multicollinearity check showed no evidence of multicollinearity after using the Variance Inflation Factor (VIF). Both bivariate and multivariable logistic regression modelling were done. Reference categories were informed by previous studies and a priori. Crude odds ratios (cOR) and adjusted odds ratios (aOR) with 95% confidence intervals (CI) were estimated. Sampling weight was assigned at various levels of the analysis to account for over- and under-sampling of some areas within the study settings while the svy command was used to take account of the complex sampling procedure. Data were processed and analyzed using Stata Version 14.0 and statistical significance was declared at *p* < 0.05.

### Ethics approval

ICF Institutional review board approved the survey. Informed consent was obtained from all the respondents before the commencement of interviews with each respondent. Further information about the DHS data usage and ethical standards are available at http://goo.gl/ny8T6X.

## Results

**Fig. 1:**
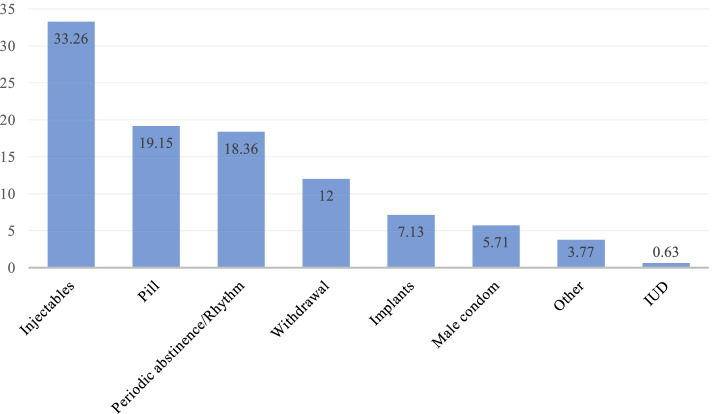
Discontinuation of contraception by method in PNG

**Fig. 2:**
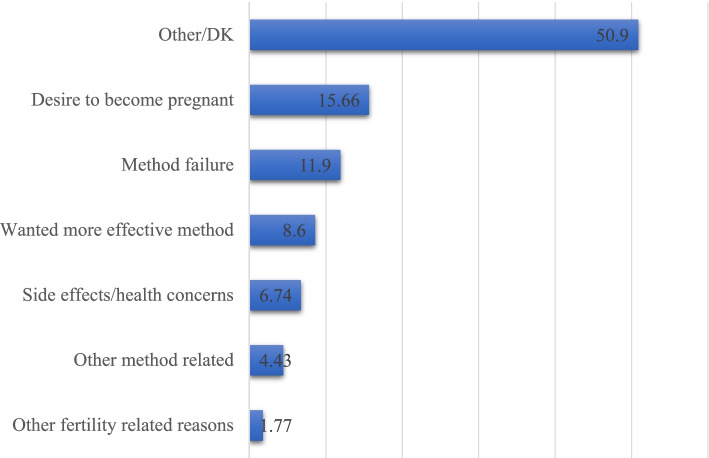
Reasons for contraception discontinuation in PNG

From Fig. 1, 33.26% of the women discontinued injectables, 19.15% discontinued pills and 3.77% discontinued other contraception methods. The major reason for discontinuing contraception were other factors which were not specified and those who did not cite a reason. Aside that, desire to become pregnant (15.66%) and method failure (11.9%) were cited as the main reasons (see Fig. 2).

### Socio-demographic, reproductive and obstetric characteristics of reproductive age women in Papua New Guinea

Table 1 shows the socio-demographic, reproductive and obstetric characteristics of reproductive age women in PNG. Approximately 26% of the women were aged 25–29. More than half (78.8%) of the respondents were married while 50.9% had primary level of education. Majority (84%) lived in the rural area. The highest proportion of the respondents (61.8%) were not working and those in the richest wealth index constituted 26.2%. The highest proportion (34.8%) were in Highlands Region. Also, 75.9% were not exposed to family planning messages but 79% had good knowledge on family planning. Again, 55.9% had no information on choice. Those who made decisions for using contraceptives jointly had the highest proportion (46.8%) and 48.8% had one birth in the last 5 years. Those who had more than four living children accounted for 38.4% while the desire for no more children accounted for 38.8%.

### Factors associated with contraceptive discontinuation among women in Papua New Guinea

The regression analysis showed that women aged 20–24 [aOR = 2.12, CI = [= [1.04,4.31] through to those aged 30–34 [aOR = 1.98, CI = 1.03,3.79] had higher odds to discontinue contraceptive usage compared to those aged 45–49. Women with no information on choice of contraception [aOR = 2.85, CI = 2.31,3.51], those with two or more births in the last five years [aOR = 2.35, CI = 1.65,3.35] and those living in the Highland region [aOR = 1.71, CI = 1.28,2.29] were more likely to discontinue contraceptive usage compared with those with information on contraceptive choices, those with no births and those living in the Island region respectively. However, women in the rural areas [aOR = 0.78, CI = 0.61,0.99], those who are undecided in their desire for more children [aOR = 0.61, CI = 0.37,1.00], women using LARC [aOR = 0.10, CI = 0.06,0.15], injectables [aOR = 0.43, CI = 0.30,0.63] and other modern methods including condom [aOR = 0.22, CI = 0.15,0.34] were less likely to discontinue contraceptive usage (Table 2).

**Table 2:** Bivariate and multivariate analysis for contraceptive discontinuation among reproductive age women in Papua New Guinea, 2016-2018 PGDHS (weighted)

Variable	Contraceptive discontinuation	
	**cOR**	**aOR**
**Age in 5-year groups**		
15–19	1.89 [0.79,4.51]	1.01 [0.39,2.61]
20–24	3.69*** [1.92,7.11]	2.12* [1.04,4.31]
25–29	3.81*** [2.03,7.16]	2.30* [1.19,4.47]
30–34	3.10*** [1.66,5.81]	1.98* [1.03,3.79]
35–39	2.27* [1.19,4.32]	1.71 [0.89,3.28]
40–44	1.68 [0.85,3.34]	1.33 [0.66,2.69]
45–49	1 [1.00,1.00]	1 [1.00,1.00]
**Marital status**		
Living together	1.03 [0.67,1.56]	0.75 [0.47,1.20]
Married	1.74** [1.24,2.44]	1.1 [0.76,1.60]
Never married/widowed/divorced/separated	1 [1.00,1.00]	1 [1.00,1.00]
**Highest educational level**		
No education	0.48* [0.26,0.90]	0.62 [0.30,1.28]
Primary	0.6 [0.33,1.09]	0.76 [0.39,1.47]
Secondary	0.61 [0.34,1.13]	0.83 [0.43,1.58]
Higher	1 [1.00,1.00]	1 [1.00,1.00]
**Residence**		
Urban	1 [1.00,1.00]	1 [1.00,1.00]
Rural	0.81* [0.67,0.98]	0.78* [0.61,0.99]
**Occupation**		
Not working	0.93 [0.76,1.15]	
Working	1 [1.00,1.00]	
**Wealth index**		
Poorest	1 [1.00,1.00]	
Poorer	1.05 [0.75,1.47]	
Middle	0.95 [0.67,1.34]	
Richer	0.93 [0.68,1.28]	
Richest	0.95 [0.67,1.35]	
**Region**		
Southern region	1.03 [0.83,1.28]	1.23 [0.95,1.59]
Highlands region	1.49** [1.15,1.94]	1.71*** [1.28,2.29]
Momase region	0.70** [0.54,0.91]	0.85 [0.63,1.14]
Islands region	1 [1.00,1.00]	1 [1.00,1.00]
**Exposure to family planning messages**		
No	0.8 [0.63,1.00]	
Yes	1 [1.00,1.00]	
**Knowledge of family planning**		
Poor	1 [1.00,1.00]	
Good	1.25 [0.99,1.57]	
**Information on choice**		
No	2.45*** [2.01,2.99]	2.85*** [2.31,3.51]
Yes	1 [1.00,1.00]	1 [1.00,1.00]
**Births in the last five years**		
None	1 [1.00,1.00]	1 [1.00,1.00]
1	1.3 [0.97,1.75]	1.07 [0.77,1.50]
2 +	2.93*** [2.15,3.99]	2.35*** [1.65,3.35]
**Number of living children**		
None	1 [1.00,1.00]	1 [1.00,1.00]
1	1.3 [0.69,2.46]	1.12 [0.56,2.27]
2	1.89* [1.02,3.51]	1.11 [0.54,2.28]
3	1.6 [0.86,3.00]	0.89 [0.43,1.87]
4 +	1.29 [0.70,2.37]	1.06 [0.50,2.26]
**Desire for more children**		
Wants within 2 years	1 [1.00,1.00]	1 [1.00,1.00]
Wants after 2 + years	0.95 [0.59,1.51]	0.86 [0.52,1.43]
Wants no more children	0.9 [0.59,1.38]	0.96 [0.59,1.58]
Undecided	0.53** [0.34,0.82]	0.61* [0.37,1.00]
**Method**		
LARC	0.09*** [0.06,0.13]	0.10*** [0.06,0.15]
Pills	1 [1.00,1.00]	1 [1.00,1.00]
Injectables	0.44*** [0.32,0.62]	0.43*** [0.30,0.63]
Other modern methods including condom	0.16*** [0.11,0.24]	0.22*** [0.15,0.34]
Traditional method	0.82 [0.59,1.16]	0.9 [0.61,1.33]
pseudo R-sq		0.197

Exponentiated coefficients; 95% confidence intervals in brackets

*cOR* Crude odds ratio, *aOR* Adjusted odds ratio

^*^p < 0.05, **p < 0.01, ***p < 0.001

## Discussion

PNG has one of the lowest contraceptive prevalence rates in the Western Pacific Region which makes contraceptive discontinuation in this country a critical public health issue worth studying [21]. This study, therefore, assessed the factors associated with contraceptive discontinuation among women of reproductive age in PNG, which is crucial in enabling family planning programs to identify appropriate strategies to improve continuous contraceptive utilization [9, 24].

We found that about 56.18% of reproductive age women in PNG discontinue contraceptive use which is alarming considering the fact that globally, it is projected that only about 38% of contraceptive users discontinue use of a method within the first 12 months [8]. This finding is also disturbing because Jain et al. [8] have further reported that findings from a survey involving reproductive age women from 34 low and middle income countries (LMICs) shows that 38% of women with unmet need of family planning were prior contraceptive method users who had discontinued use. Considering that the Oceania region has one of the lowest contraceptive prevalence globally (57.8%) [25], a higher prevalence of discontinuation is a major source of worry. The discontinuation prevalence found in this study is also higher than those reported in other LMICs such as Bangladesh [26], Kenya [24], Ethiopia [4], Zimbabwe, Armenia, Egypt and Colombia [27]. The plausible reasons could be inadequate information on choice, low socioeconomic status, sociocultural factors, high illiteracy levels of many reproductive aged women coupled with many barriers women face in accessing healthcare including family planning services in PNG [16, 28–30].

With regards to method specific discontinuation, it is not surprising that in this study about 33.26% of women discontinued using the injectables as it is reported in a previous study that the injectables is the most widely used contraceptive method in PNG [28]. The finding that injectables is widely the most discontinued contraceptive method among women of reproductive age is also corroborated by previous studies in Nigeria [31] and Bangladesh [26]. Injectables could be short term and may not require medical intervention (Health professionals) to discontinue unlike the implant and IUD. This probably makes it easier for women to discontinue. However, we found that reproductive age women who were using LARC, injectables and other modern methods including condom were less likely to discontinue. The plausible reason could be that women who were still using contraceptives have been using these methods for a while and they know the benefits associated with its use and so discontinuation becomes a disincentive.

Findings from this study further shows that women living in the Highlands region had higher odds of contraceptive discontinuation. This could possibly be attributed to high prevalence of poor utilization of family planning services and high prevalence of low knowledge about family planning services among both men and women in the Highlands region of PNG [30]. Comprehensive knowledge about FP is often a pathway to women’s empowerment over their reproductive health and holistic wellbeing which increases awareness of benefits and de-merits of contraceptives and subsequently aiding their decision making. Our finding is substantiated by findings from a previous study in PNG by Seidu et al. [28]. In the Highlands region, the odds of contraceptive uptake is high but poor knowledge and inadequate information on choice coupled with poor family planning services or access could plausibly explain the high contraceptive discontinuation rates.

Consistent with previous studies [32, 33], we also observed that reproductive aged women in PNG with no information on choice were more likely to discontinue contraceptive use compared to their counterparts who had information. Timely and accurate information is key with regards to contraceptive uptake. Information on choice empowers women to select the method that best satisfies their personal, reproductive and health needs based on a thorough understanding of their contraceptive options [34]. When women are well informed to make their own decision, it increases their confidence and commitment to their health care decisions and thereby reducing the odds of discontinuation. Women of reproductive age are more likely to use contraceptive and stay with it if they receive adequate information on choice.

Furthermore, women with two or more parity within the last 5 years preceding the DHS were more likely to discontinue contraceptive use within the first 12 months. This finding is consistent with findings from studies in other LMICs [4, 13, 22, 35]. The desire to have more children is the motivation for contraceptive discontinuation for some women. For example, in Indonesia, a study reported that women who wanted more children had lower odds of using contraceptives compared to those who reported not wanting more children [36]. Generally, women may discontinue contraceptive use immediately they develop the desire to have more children and easy to discontinue methods such as condoms, pills and injectables only exacerbate the situation.

## Strength and limitations of the study

Causal inferences cannot be made from this study because of the cross-sectional study design that was employed. Also, due to the retrospective nature of the study, there is the potential for recall bias. Nevertheless, this study provides a nationally representative coverage of contraceptive discontinuation among women of reproductive age in PNG.

## Conclusion and implications for PNG

We found that about 56.18% of reproductive age women in PNG discontinue contraceptive use within the first 12 months. Also, the odds of discontinuation were higher among women residing in the highlands, those with no information on choice and those with two or more births within the last five years preceding the 2016-2018 PGDHS. However, those who were using LARC, injectables and other modern methods including condom were found to be less likely to discontinue. The findings of this study has several multifaceted implications for PNG.

To reduce the prevalence of contraceptive discontinuation in PNG, the National Department of Health (NDOH) in PNG needs to intensify education on the benefits of contraceptives and choice. A nationwide mass education on contraceptives will empower women with enough information to overcome misconceptions and misunderstandings thereby making discontinuation unattractive and a disincentive. The NDOH should develop a nationwide comprehensive communication and advocacy strategy that will be implemented at all levels including local levels and in the Highland Regions. The nationwide education should pay critical attention to long term methods such as the IUD and LARC which often requires health professional intervention to either use or discontinue so that women who may want to discontinue as a result of misinformation or misconceptions could be counseled and helped. Paying special attention to these methods is also important because in this study, we observed that reproductive aged women who were using LARC and IUD were less likely to discontinue their use. The nationwide mass education on the benefits of contraceptives will also solve the issue with many reproductive aged women lacking information on choice and discontinuing with contraceptive usage. The nationwide mass education should also pay attention to region specific contraceptive and family planning needs. For example, in this study we observed that contraceptive discontinuation was high among women living in the highlands region. The PNG National Health Information System also needs to be updated to include implant insertions especially as findings in this study have highlighted that women using this method are less likely to discontinue. Adequate information on all the methods will help women to make informed choices and empower them to dispel misconceptions that often robs them of the true benefits of contraceptives.

## Data Availability

The dataset supporting the conclusions of this article is available online at https://dhsprogram.com/data/dataset/PapuaNewGuinea

## Abbreviations

aOR: Adjusted Odds Ratio
CI: Confidence Interval
CI: Confidence intervals
cOR: Crude odds ratios
DHS: Demographic and Health Survey
EA: Enumeration areas
FP: Family planning
ICF: Inner City Fund
IUD: Intra Uterine Device
LMICs: Low-and-middle-income countries
PGDHS: Papua New Guinea Demographic and Health Survey
PNG: Papua New Guinea
SDG: Sustainable Development Goals
VIF: Variance Inflation Factor
